# Comparative Analysis of Hulless Barley Transcriptomes to Regulatory Effects of Phosphorous Deficiency

**DOI:** 10.3390/life14070904

**Published:** 2024-07-19

**Authors:** Likun An, Ziao Wang, Yongmei Cui, Yixiong Bai, Youhua Yao, Xiaohua Yao, Kunlun Wu

**Affiliations:** 1 Academy of Agriculture and Forestry Sciences, Qinghai University, Xining 810016, China; anlikun@163.com (L.A.); 13043832262@163.com (Z.W.); 15251892177@163.com (Y.C.); yixiongbai@163.com (Y.B.); 2010990021@qhu.edu.cn (Y.Y.); 2009990027@qhu.edu.cn (X.Y.); 2 Laboratory for Research and Utilization of Qinghai Tibet Plateau Germplasm Resources, Xining 810016, China; 3 Qinghai Key Laboratory of Hulless Barley Genetics and Breeding, Xining 810016, China; 4 Qinghai Subcenter of National Hulless Barley Improvement, Xining 810016, China

**Keywords:** hulless barley, P deficiency, DEGs, GO annotation, KEGG pathway, gene expression

## Abstract

Hulless barley is a cold-resistant crop widely planted in the northwest plateau of China. It is also the main food crop in this region. Phosphorus (P), as one of the important essential nutrient elements, regulates plant growth and defense. This study aimed to analyze the development and related molecular mechanisms of hulless barley under P deficiency and explore the regulatory genes so as to provide a basis for subsequent molecular breeding research. Transcriptome analysis was performed on the root and leaf samples of hulless barley cultured with different concentrations of KH_2_PO_4_ (1 mM and 10 μM) Hoagland solution. A total of 46,439 genes were finally obtained by the combined analysis of leaf and root samples. Among them, 325 and 453 genes had more than twofold differences in expression. These differentially expressed genes (DEGs) mainly participated in the abiotic stress biosynthetic process through Gene Ontology prediction. Moreover, the Kyoto Encyclopedia of Genes and Genomes showed that DEGs were mainly involved in photosynthesis, plant hormone signal transduction, glycolysis, phenylpropanoid biosynthesis, and synthesis of metabolites. These pathways also appeared in other abiotic stresses. Plants initiated multiple hormone synergistic regulatory mechanisms to maintain growth under P-deficient conditions. Transcription factors (TFs) also proved these predictions. The enrichment of ARR-B TFs, which positively regulated the phosphorelay-mediated cytokinin signal transduction, and some other TFs (AP2, GRAS, and ARF) was related to plant hormone regulation. Some DEGs showed different values in their FPKM (fragment per kilobase of transcript per million mapped reads), but the expression trends of genes responding to stress and phosphorylation remained highly consistent. Therefore, in the case of P deficiency, the first response of plants was the expression of stress-related genes. The effects of this stress on plant metabolites need to be further studied to improve the relevant regulatory mechanisms so as to further understand the importance of P in the development and stress resistance of hulless barley.

## 1. Introduction

Hulless barley is widely distributed in the Tibetan cluster area in northwest and southwest China, which all belong to high-elevation regions. As the main staple food crop in Tibet, hulless barley is still the dominant food among Tibetans. Natural phytochemicals can promote people’s health, especially because they have antidiabetic activity, which is due to their high contents of β-glucan, phenolics, and flavonoids [1]. Hulless barley has the characteristics of cold tolerance, barren tolerance, low-temperature tolerance, and strong drought resistance. Moreover, hulless barley is the only grain crop that can mature normally at an altitude of 4200 m [2]. The “Du Lihuang” highland barley as the research subject is extensively cultivated in Qinghai, Tibet, and other major highland barley planting regions in China. It is a precocious variety and reaches a height of 75–95 cm. This variety demonstrates strong tillering ability and produces abundant grains on its panicles. Moreover, it possesses remarkable resistance to waterlogging, cold weather conditions, light hail damage, and drought stress and exhibits resistance against diseases and pests. Furthermore, this variety displays adaptability to various environments resulting in high yields per unit area.

As one of the macronutrients, phosphorous (P) is related to plant growth and development [3]. It is an important essential nutrient and the structural and functional component of nucleic acids, nucleotides, phospholipids, and high-energy molecules (ADP and ATP), and it is an activated intermediate in the photosynthetic carbon cycle. In addition, inorganic phosphate (Pi) also plays an important role in metabolism, protein regulation, and signal transduction cascades [4]. However, the Pi concentration is extremely low in soil and easily forms insoluble complexes, which is because of its prospect of binding strongly to soil surfaces [5]. However, plants can only use less than 30% of the Pi fertilizer, and the rest is lost in the environment, leading to soil degradation and the eutrophication of water bodies [6]. The lack of phosphorus (P) can inhibit cell development, resulting in reduced seed or fruit growth and even lower yields. Approximately five billion hectares of farmland are short of available P, which requires an annual 2% increase to maintain the current food production [7].

The phenomenon of the P shortage and high cost can be resolved by enhancing the utilization of P in crops through genetic improvement technology, which is the key to viable, sustainable yield production [8]. However, plants have evolved complex responses and adaptive mechanisms to preserve the development and homeostasis in the absence of soil P [9]. Nevertheless, the relevant molecular mechanisms and regulatory elements have only been verified and analyzed in some crops [10]. Phosphate transporters (PHTs) are a group of structurally related proteins that mediate the transmembrane transport of organic anions under low-P stress [11]. Moreover, PHT1 mainly participates in root-mediated P uptake from the soil in *Arabidopsis* under low P conditions. Other PHT1 homologous transporters also play crucial roles in different parts of plants [12]. After being absorbed by the root cells, phosphate1 (PHO1) can translocate P from the root to the shoot and load to the xylem [13]. Moreover, some members in the WRKY transcription factors (TFs) can promote PHT1 expression [14] and coordinately inhibit PHO1 expression [15]. Although relevant genes have been reported in model plants, more detailed and intensive research is still under way, especially in other crops.

In the present study, hulless barley cultivar “Du Lihuang” (ZDM01467) was used to investigate the change in response to low-P stress. The study also aimed to disclose the mechanisms of low-P tolerance and identify the relevant candidate genes through transcriptome analysis.

## 2. Materials and Methods

### 2.1. Plant Growth

The hulless barley seeds “Du Lihuang”, which was the early maturing diploid variety cultivated in Qinghai, were disinfected with sodium hypochlorite for 8 min, washed with clean water, and placed in the culture dish for germination at 25 °C under continuous light. After germination, the seedlings with similar growth potential were selected and fixed on the floating plate. Six plants of each kind of hulless barley were placed in two plastic boxes (600 × 500 × 160 mm^3^). The modified Hoagland medium (1 mmol/L KH_2_PO_4_ as the only source of element P) was used for culture; 20 L of Hoagland solution was added to each box under a light/dark cycle of 16 h/8 h at 25 °C/18 °C. When the plant grew to the 3-leaf stage with endosperm nutrient depletion, the medium in one box was changed to low-P-modified Hoagland medium (10 μmol/L KH_2_PO_4_). The physiological indexes of hulless barley, including shoot height, root length, fresh weight of shoots and roots, dry weight of shoots and roots, total P content, soluble sugar content, protein content, free proline content, MDA content, and SOD, POD, CAT, and ACP activities, were measured when the plants grew to the five-leaf stage.

### 2.2. RNA Extraction, Library Construction, and Sequencing

The total RNA was extracted using a TRIzol reagent kit (Invitrogen, Carlsbad, CA, USA). The RNA quality was assessed on an Agilent 2100 Bioanalyzer (Agilent Technologies, Santa Clara, CA, USA) after extracting the total RNA. Then, the enriched mRNA was fragmented into short fragments and reverse-transcribed into cDNA using the NEBNext Ultra RNA Library Prep Kit for Illumina (New England Biolabs, Ipswich, MA, USA). The purified double-stranded cDNA fragments were end repaired, a base was added, and ligated to Illumina sequencing adapters. The ligation reaction was purified with the AMPure XP Beads (1.0×). The ligated fragments were subjected to size selection by agarose gel electrophoresis and PCR amplified. The resulting cDNA library was sequenced using Illumina Novaseq6000 (Gene Denovo Biotechnology Co., Guangzhou, China).

### 2.3. Alignment with a Reference Genome

An index of the reference genome was built, and paired-end clean reads were mapped to the reference genome using HISAT 0.1.6 [16] and other parameters set as a default. The mapped reads of each sample were assembled using StringTie [17] in a reference-based approach. For each transcription region, an FPKM value was calculated to quantify its expression abundance and variations, using the RSEM v1.3.3 software [18].

### 2.4. Differentially Expressed Genes

The RNA differential expression analysis was performed using the DESeq2 1.25.9 [19] software between two different groups and using the edgeR 3.32.1 software [20] between two samples. The genes/transcripts with a false discovery rate below 0.05 and absolute fold change ≥2 were considered differentially expressed genes/transcripts. GO [21] is an international standardized gene functional classification system that offers a dynamic-updated controlled vocabulary and a strictly defined concept to comprehensively describe the properties of genes and their products in any organism. GO has three ontologies: MF, CC, and BP. Each GO belongs to a type of ontology. The GO enrichment analysis showed that all GO terms were significantly enriched in DEGs compared with the genome background, and the DEGs that corresponded to biological functions were filtered. All DEGs were mapped to GO terms in the GO database (http://www.geneontology.org/); gene numbers were calculated for every term, and significantly enriched GO terms in DEGs compared with the genome background were defined using the hypergeometric test. Genes usually interact with each other to play roles in certain biological functions. The pathway-based analysis helped further understand the biological functions of genes. KEGG [22] is the major public pathway-related database. Pathway enrichment analysis identified significantly enriched metabolic pathways or signal transduction pathways in DEGs compared with the whole-genome background.

### 2.5. Gene Set Enrichment Analysis

We performed GSEA using the software GSEA v1.0 and MSigDB v7.5.1 [23] to identify whether a set of genes in specific GO terms/KEGG pathways showed significant differences in the two groups. Briefly, we input gene expression matrix and rank genes using the signal-to-noise normalization method. The enrichment scores and *p* values were calculated in default parameters.

### 2.6. Validation and Analysis of DEGs Using qRT-PCR

The qRT-PCR analysis was performed using the Bio-Rad Real-Quantitative real-time PCR analysis Time System (CFX96, BioRad, Hercules, CA, USA). The primers were designed using Primer Premier 6. The gene-specific primer sequences for qRT-PCR are listed in Table S14. We use a 96-well Polypropylene Flat Top PCR Microplate, Low Profile, No Skirt, Clear, Nonsterile (PCR-96-LP-FLT-C, Axygen, Union City, CA, USA) for the qPCR reaction. The qPCR reaction system (20 μL) was as follows: forward primers 1 μL, reverse primers 1 μL, cDNA (500 ng/μL) 2 μL, THUNDERBIRD SYBR qPCR Mix 10 μL, ddH_2_O 6.0 μL, totally 20 μL. The amplification procedure is predenaturation 95 °C 30 s; denaturation 95 °C 5 s, annealed 55 °C 20 s, extended 72 °C 30 s, 40 cycles. We performed three technical repeats and three independent biological replicates and used the most stable gene 18S ribosomal RNA as the reference gene in qRT-PCR analyses [24]. Quantitative analysis and statistics were performed using the 2^−ΔΔCt^ method [25].

## 3. Results

### 3.1. Analysis of Hulless Barley Morphology under Low-P Treatment

**Figure 1:**
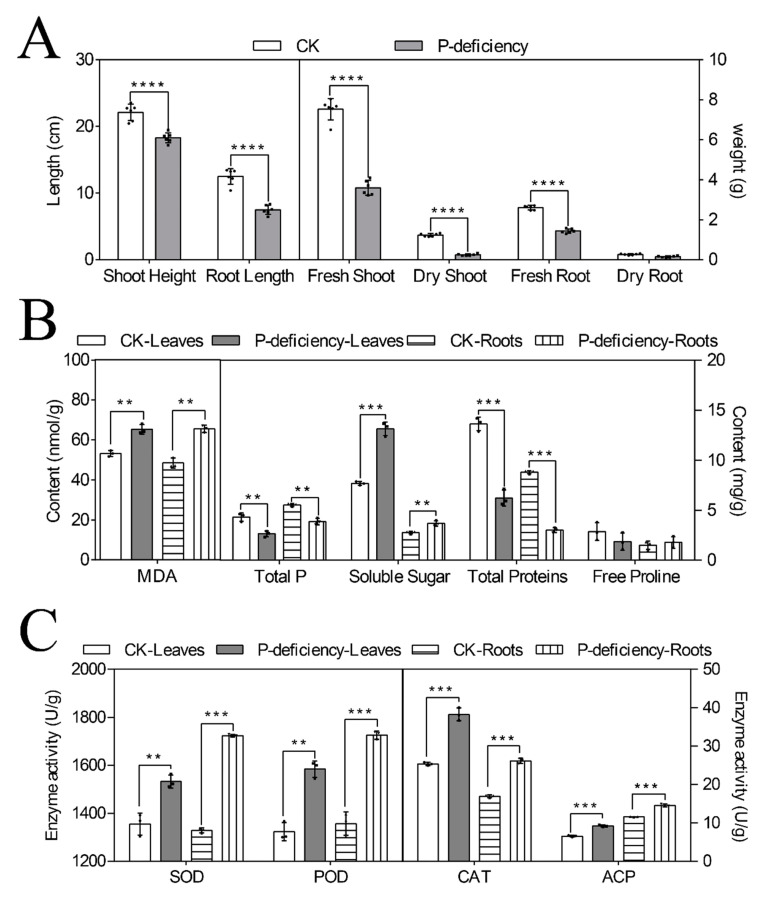
Difference in the phenotype (**A**), endogenous content (**B**), and enzymatic activity (**C**) under P deficiency (**** indicates *p* < 0.001, *** indicates *p* < 0.01, and ** indicates *p* < 0.05).

The phenotypic differences of the hulless barley cultivar “Du Liang,” which included shoot height, root length, fresh weight of shoots and roots, dry weight of shoots and roots, and root–shoot ratio, were measured under the low-P treatment (Figure 1A and Table S1). The results showed that the hulless barley cultivar “Du Liang” was significantly sensitive to the P level. The lower-P treatment limited growth and biomass accumulation in plants (Figure 1A), and the values of the root–shoot ratio calculated under different treatments were opposite to those for the growth potential.

In addition, the endogenous content (Figure 1B and Table S1) and enzymatic activity assayed in leaves and roots (Figure 1C and Table S1) showed that the total P, total proteins, and free proline contents reduced and soluble sugar and malondialdehyde (MDA) contents increased under low-P treatment. The superoxide dismutase (SOD), peroxidase (POD), catalase (CAT), and acid phosphatase (ACP) activity were all enhanced under low-P. Interestingly, all values were higher in the leaves than in the roots, except for the total P content, which was different for the different P concentrations (Figure 1B,C).

### 3.2. Database Quality and Mapping Gene Analysis by RNA-seq

**Figure 2:**
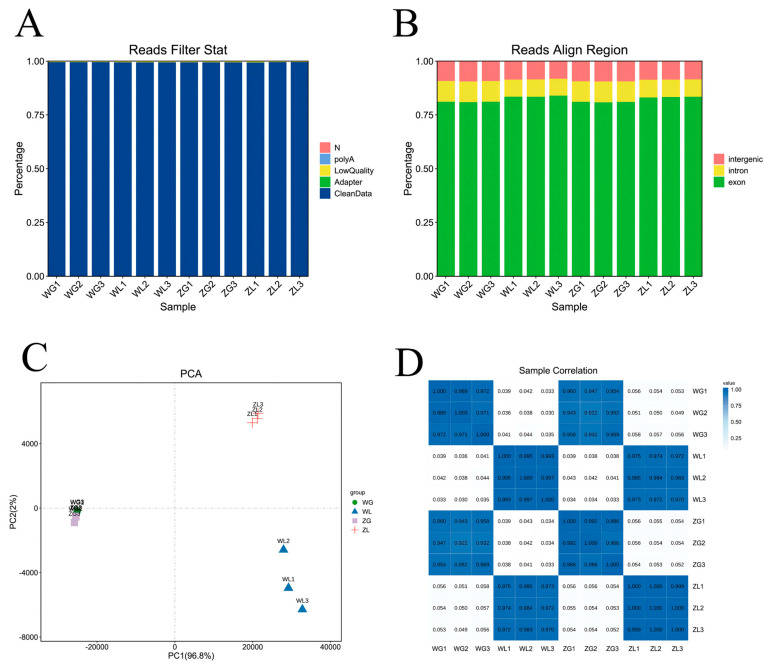
Database quality and mapping gene analysis using RNA-seq. (**A**) Read filter stat in each sample; (**B**) the coverage of genome alignment and gene location; (**C**) relationship between samples (Figure S4); (**D**) the cluster analysis of the replicates within the treatment.

We used transcriptome sequencing technology to detect and analyze the samples to comprehensively understand the molecular regulatory mechanism of “Du Liang” under the low-P treatment. The RNA-seq databases (all uploaded in the NCBI database, which is shown in File S1), containing roots and leaves in normal- and low-P treatments, were used to acquire clean reads, which all accounted for more than 99.4% in each sample (Figure 2A and Table S2). Each clean data contained more than 6 billion bases, and even reached 7.4 billion. Nevertheless, only less than 6.3% of bases had the probability of false identification (Q30 database in Table S2). The GC content was approximately 50% and the average base sequencing quality was nearly 40, implying that the composition and distribution of bases were of high quality, providing a good data source for the subsequent analysis.

The coverage of the genome alignment of leaf samples reached more than 93.7% and the root samples covered more than 72.5% (Table S3). The analysis of the total mapped reads results in each sample indicated that more than 80.8% of the reads were blasted in exons, and about 9% were located in introns and intergenic regions (Figure 2B and Table S3). The analysis of the blasted genes showed that 3588 genes were defined as novel genes and referred to about 107 plant signal regulation and synthesis pathways (Table S4). Moreover, these databases provided a basis for the following analyses.

The original read count data were corrected to obtain more accurate fragment per kilobase of transcript per million mapped reads (FPKM) expression data to further improve the accuracy of gene expression (Table S5). Combined with these data, the analysis of the relationship between samples showed that the data of leaf samples (WL and ZL) and root samples (WG and ZG) were significantly different; also, obvious differences were found between the leaf samples under the low-P (WL) and normal-P (ZL) conditions. The root samples were very small and basically clustered together (Figure 2C). The cluster analysis of the replicates within the treatment showed that, except for the small differences among the leaf samples under the low-P treatment, basically no differences were found among the other samples with good repeatability (Figure 2D).

#### Analysis of DEGs and TFs

**Figure 3:**
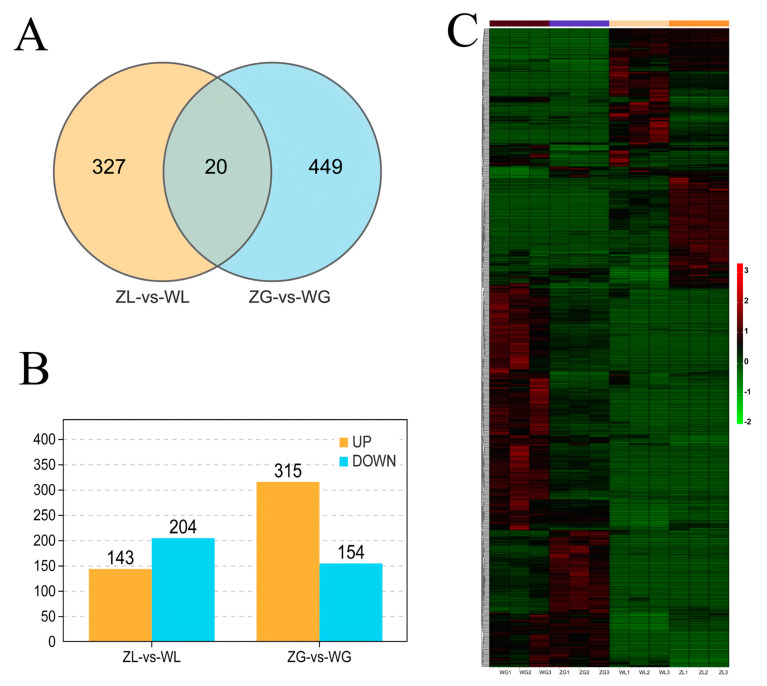
Analysis of DEGs. The Venn diagram (**A**) shows the number of DEGs and co-responsive genes in the leaf and root samples. The upregulated or downregulated DEGs were also counted (**B**), and these gene expression patterns are shown in the heatmap (**C**) (Table S16).

The analysis of gene expression in samples treated with different P concentrations showed that 325 and 453 genes were differentially expressed by more than twofold in leaf and root samples, respectively. Moreover, 20 genes responded to the regulation of the P element in both leaf and root tissues (Figure 3A and Table S5). The comparative analysis of the gene expression data also showed that 132 genes were upregulated and 193 were downregulated in the leaf expression data, and 300 were upregulated and 153 were downregulated in the root expression data (Figure 3B).

In this study, we found 1763 TFs from the whole genes, which belonged to 55 families (Figure S1 and Table S6). Differential analysis showed that only 58 TFs were DEGs, which is two times less than under low-P treatment compared with the normal-P treatment. Further, 17 and 44 TFs belonged to the leaf and root samples, respectively. Also, three TFs were present in both leaf and root samples. Among these, 7 TFs were downregulated and 10 TFs were upregulated in the leaf samples; and 7 TFs were downregulated and 44 TFs were upregulated in the root samples. These differentially expressed TFs belonged to 12 TF families, which contained 5 and 12 families in the leaf and root samples, respectively (Table S6).

### 3.3. Classification of GO Functional Annotations and KEGG Pathways for DEGs

**Figure 4:**
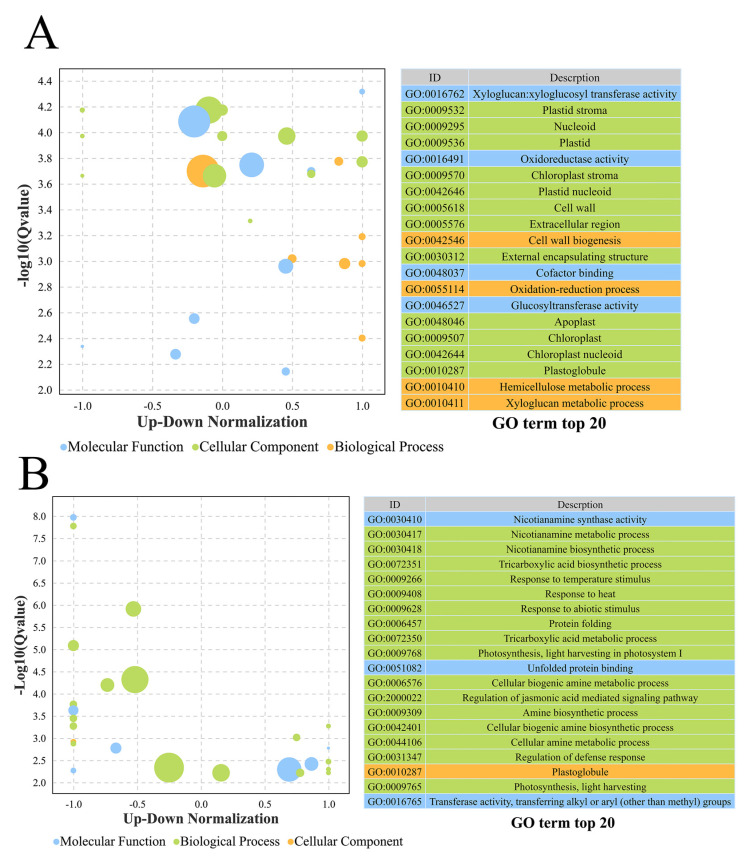
Classification of GO functional annotations for DEGs. (**A**) indicates the ZL/WL and (**B**) indicates the ZG/WG. The circles size indicated the enrichment of gene numbers.

The GO annotations were predicted to further analyze the function of DEGs. These genes were mainly divided into three categories, including biological process (BP), cell component (CC), and molecular function (MF). The genes were enriched in the metabolic and cellular processes in BP, cells and cell parts in CC, and binding and catalytic activities in MF in the leaf samples. Except for these annotations, the root DEGs also participated in membrane and membrane parts in CC in different treatment samples (Figure S2). In addition, we analyzed the GO annotations by twofold DEGs, and the top 20 enriched GO terms were displayed by q values (Figure 4). A total of seven BP, nine CC, and four MF annotations were relatively more accurate in gene structure comparisons in leaf DEGs. These annotations were mainly clustered in cell wall-related BPs, plasmids, and oxidation–reduction functions; also, a small number of them were related to the xyloglucan metabolic process (Figure 4A and Table S7). The analysis of the results of the top 20 GO annotations enriched in root samples revealed that the participating functions/processes of their DEGs were different from those in leaves. Then, 15 BPs, 1 CC, and 4 MFs were clustered, which mainly participated in abiotic stresses, the nicotianamine-related bioprocess, and biogenic amine biosynthetic process. Some signaling pathways, transferase activities, and other annotations were also predicted to be involved in genes (Figure 4B and Table S8).

**Figure 5:**
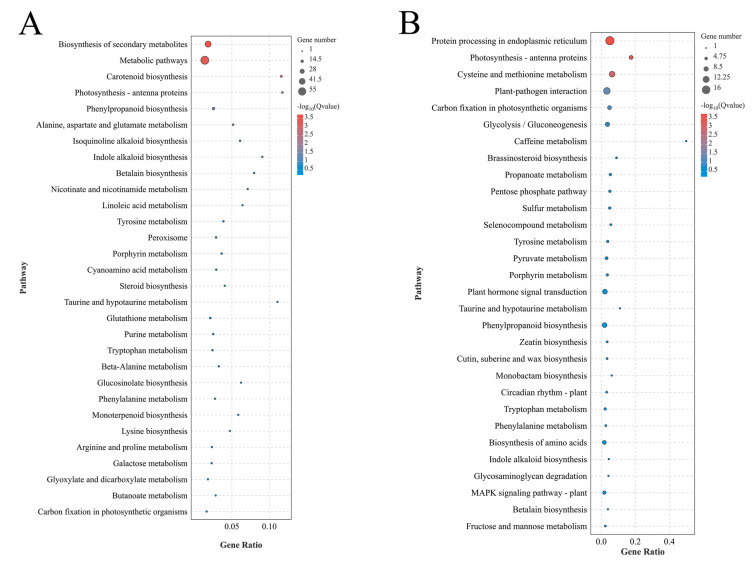
Classification of KEGG pathways for DEGs. (**A**) indicates the ZL/WL and (**B**) indicates the ZG/WG.

The use of the KEGG database to compare and analyze the DEGs and the relevant data showed that the DEGs were mainly clustered as “Global and overview maps” in metabolism. Compared with the blasted data of two different tissues, “Environmental adaptation” in organismal systems was significant and more DEGs were clustered in “Folding, sorting, and degradation” in the genetic information processing and in “Signal transduction” in the environmental information processing in root samples (Figure S3). The top 20 KEGG pathways by DEGs were mainly clustered in the biosynthesis of secondary metabolites and metabolic pathways under the P treatments in leaves (Figure 5A and Table S9). In the root tissues, the DEGs focused on protein processing in the endoplasmic reticulum, cysteine and methionine metabolism, and plant–pathogen interaction (Figure 5B and Table S9). Similar to the GO annotations, a significant difference was found between the KEGG pathway of root and leaf DEGs. Thus, the results suggested that the differential expression of functional genes might affect multiple metabolic pathways in different tissues.

### 3.4. Gene Set Enrichment Analysis in Whole-Expression Genes

The gene set enrichment analysis (GSEA) uses all genes, rather than just DEGs, to identify functional gene sets that are not significantly different but have similar differential expression trends and to determine whether the corresponding pathways are activated or repressed. A total of 441 GO annotations were enriched in leaf treatments. Moreover, 365 GOs were upregulated in the ZL samples and 76 GOs were upregulated in the WL samples. A total of 223 GOs were enriched in root treatments. Then, 200 GOs were upregulated in the ZG samples and 23 in the WG samples. Contrary to leaf treatments, the higher enrichment score was clustered in the ZG samples, which mainly included the photosystem, transport- and binding-related functions, protein structure regulation, and other functions. Further, 76 GOs existed in leaf and root treatments simultaneously (“go.both” sheet in Table S10). These GOs were involved in a lot of functions.

The KEGG pathway by GSEA enrichments showed that 28 pathways were mainly enriched in leaf treatments and 31 in root treatments. A total of 4 KEGG pathways were upregulated in WL and 24 in ZL in leaf treatments, and 15 were upregulated in WG and 16 in ZG in root treatments. Alkaloid biosynthesis, ester metabolism, and nucleotide regulation were aligned in the leaf samples, and photosynthesis, hormone transduction, plant–pathogen interaction, biosynthesis, amino acid metabolism, and other modifications were aligned in the root samples (Table S10). A total of 13 KEGG pathways existed in the leaf and root treatments simultaneously (“kegg.both” sheet in Table S10). These KEGG pathways were clustered in energy and amino acid metabolism, translation, and replication and repair.

### 3.5. Verification of the Expression of DEGs under P Deficiency Using qRT-PCR

**Figure 6:**
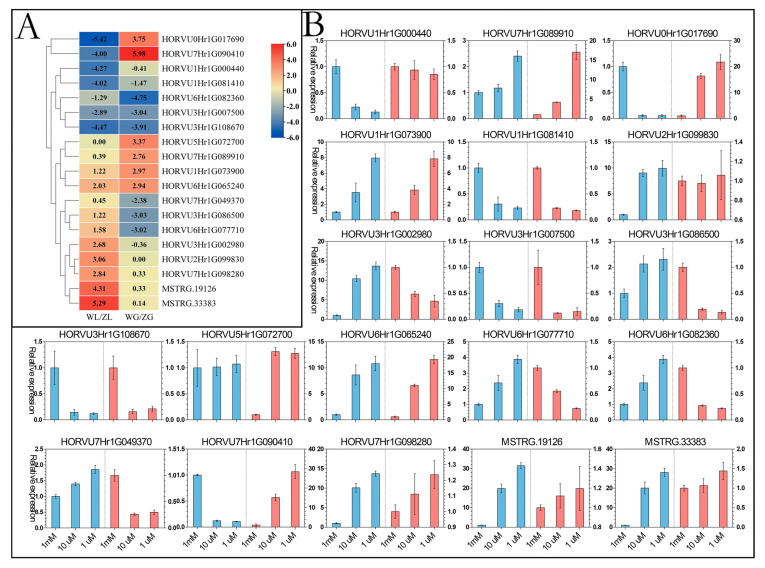
Verification of the expression of DEGs under P deficiency using qRT-PCR. The heatmap (**A**) shows the FPKM value according to RNA-seq databases, and the column charts (**B**) show the qRT-PCR results in each gene. The blue column denotes the leaf samples, and the red one denotes the root samples.

We selected 19 DEGs, which all had higher expression differences and good repeats in leaf and root samples, to identify the expression of the main DEGs. The expression pattern of 19 DEGs using the quantitative reverse transcription polymerase chain reaction (qRT-PCR) is shown in Figure 6 (Table S15). Twelve genes (*HORVU3Hr1G086500*, *HORVU1Hr1G073900*, *HORVU2Hr1G099830*, *HORVU3Hr1G002980*, *HORVU6Hr1G065240*, *HORVU6Hr1G077710*, *HORVU6Hr1G082360*, *HORVU7Hr1G049370*, *HORVU7Hr1G098280*, *MSTRG.19126*, *MSTRG.33383*, and *HORVU7Hr1G089910*) were upregulated following a decrease in the concentration. On the contrary, six genes (*HORVU0Hr1G017690*, *HORVU1Hr1G000440*, *HORVU1Hr1G081410*, *HORVU3Hr1G007500*, *HORVU3Hr1G108670*, and *HORVU7Hr1G090410*) were downregulated and no significant difference was found in the expression level of one gene (*HORVU5Hr1G072700*) in the leaf samples. Further, seven genes (*HORVU1Hr1G073900*, *HORVU3Hr1G086500*, *HORVU5Hr1G072700*, *HORVU6Hr1G065240*, *HORVU7Hr1G089910*, *HORVU0Hr1G017690*, and *HORVU7Hr1G090410*) were upregulated following a decrease in the concentration, seven genes (*HORVU1Hr1G081410*, *HORVU3Hr1G007500*, *HORVU6Hr1G077710*, *HORVU6Hr1G082360*, *HORVU7Hr1G049370*, *HORVU3Hr1G108670*, and *HORVU3Hr1G002980*) were downregulated, and five genes (*HORVU1Hr1G000440*, *HORVU2Hr1G099830*, *HORVU7Hr1G098280*, *MSTRG.19126*, and *MSTRG.33383*) had no significant differences in the root samples.

## 4. Discussion

Green revolution is a key direction of crop research, which mainly focuses on the efficient use of fertilizers [26]. In this study, we analyzed “Du Lihuang” hulless barley under the low-P treatment. The phenotypic traits and genetic correlation in roots and leaves were analyzed using RNA-seq to study genome-wide changes in gene transcription and screen existing gene resources in response to low P concentrations [27]. It is necessary to acquire the plant response in low-P treatments, especially at the physiological and transcriptomic levels, to enhance the P-use efficiency [28].

Under the P-deficiency treatment, the shoot height, root length, fresh weight of shoots and roots, and fresh weight of total roots were significantly reduced compared with those of controls (Figure 1). These phenomena also appeared in other plants [29]. Aimen et al. also reported this result from another perspective; they suggested that higher P concentration could increase the plant height and root length [30]. Moreover, the root dry weight was not obviously different under different treatments [31]. These phenomena indicated that the distribution of dry matter to roots could increase under low P concentrations and the root–shoot ratio could be higher, which was similar to the results obtained by Reddy [32]. The higher root–shoot ratio could be an adaptive strategy for increasing P acquisition under P-deficiency treatment. Liu et al. also indicated that the genotypes with higher root length could significantly enhance P absorptivity under low-P conditions [33].

Compared with other studies, the decreasing content of total P and proteins acquired the same tendencies under P-deficient conditions [34]. Yao et al. examined persistent deficiency of the P element, which led to the gradual decrease in the common bean total P content [35] and synchronously decreased the content of total proteins because P is a key synthetic substrate of proteins. Nadeem et al. found that P nutrition improved photosynthesis [36], and starch synthesis was closely related to photosynthesis and provided ATP for starch synthesis through photophosphorylation [37]. However, our results showed that the soluble sugar content was higher under P-deficient conditions, which was attributed to low sink demand and limited leaf expansion under P starvation [38]. Some other studies also reported similar results [39]. The results also indicated that low P concentration could enhance plant stress resistance and antioxidant activity. Also, the related enzyme activities (SOD, POD, and CAT) [40] and MDA content were significantly enhanced (Figure 1B,C) [38]. Under P-deficient conditions, plants synthesize and secrete ACP, which degrades organophosphorus into inorganic P or regulates cell wall structure, thus improving the adaptability of plants to P-deficiency stress [41]. Thus, the activity of ACP was obviously higher under P-deficient conditions (Figure 1C). The analysis of the morphology databases showed that P deficiency could influence plant development; on the contrary, it could enhance plant stress resistance for plant survival.

The transcriptome information can be used to analyze the phenotypic differences under P treatments. All testing samples had a higher number of clean reads, fewer false identification in Q30 databases, and high quality of base composition, indicating that we acquired high-confidence data to ensure the accuracy of subsequent analysis [42]. Plenty of clean reads were mapped to the reference genome from *Hordeum vulgare* ssp. *vulgare* L. because the cultivar “Du Liang” belonged to a branch of the barley genus. In addition, we also acquired some new genes that could provide a possibility to explore new regulatory mechanisms. In this study, we mainly focused on the expression patterns to find out the major DEGs. The sample repeats needed higher uniformity, and the sample cluster also indicated their good repeatability [43]. The study provided a good data source and an important reference for subsequent data analysis.

DEGs, as important data, could directly reflect the molecular evidence of differences between samples [44]. Compared with gene transcriptional expression and clustering, the function by DEGs and GO annotation and KEGG pathway analysis could provide candidate genes for subsequent related studies. These candidate genes might have a potential role in increasing P-use efficiency. We performed clustering and expression pattern analysis on DEGs under different treatments, which could intuitively highlight the differences in the expression of related genes under different treatments. Although certain differences existed in the expression of small parts of different samples under the same processing, the overall trend was still consistent (Figure 3C).

The results of GO annotation and KEGG pathway prediction of GSEA and DEGs showed that plenty of genes participated in various functions and multifarious pathways. A total of 272 and 360 DEGs were annotated in the GO term, and 77 and 113 DEGs might take part in different pathways. A total of 10 GOs (blue color in Table S10) were clustered compared with the top 20 GO annotations in DEGs. The higher enrichment score was clustered in WL samples, which mainly included carbon fixation, photorespiration, sugar-related modification, and fatty-acyl-CoA, which did not appear in the DEG analysis. Moreover, 13 GOs (red color in Table S10) were clustered compared with the top 20 GO annotations in DEGs. The photosystem and some transport and cell part components did not appear in DEGs. The results mainly indicated that these DEGs were involved in DNA, RNA, GTP, protein, and ATP binding; some transporter, enzyme, and amino acid activities; and sugar, glucose, and fat binding in the leaf samples (Figure S2 and Table S11) [45]. These enzymes were mostly related to stress regulation and some phosphatase-related activities [46]. Moreover, iron and metal ion binding also clustered and participated in chloroplast function and transfer [47]. More enzyme activity and transporters were predicted in the root samples compared with the leaf samples. Calcium ion binding (GO:0005509) significantly appeared in the roots, and Liu also identified the correlation of calcium ions with plant response to low-P stress [48]. A large number of DEGs were related to the carbohydrate metabolic process, oxidation–reduction process, and phosphorylation by BP. Except for phosphorylation, the DEGs were also clustered in the regulation of transcription in root samples [49]. This also indicated that the roots were more involved in the absorption and transport of P. The KEGG results were also consistent with GO annotation findings, and DEGs were involved in photosynthesis, plant hormone signal transduction, glycolysis, phenylpropanoid biosynthesis, and the synthesis of metabolites. These pathways also appeared in other abiotic stresses [50]. These databases indicated that P deficiency was closely related to stress resistance and photosynthesis. Plants initiated multiple hormone synergistic regulatory mechanisms to maintain growth under P-deficient conditions. Thus, some TFs also appeared in KEGG pathways (Figure S3 and Table S12).

TFs played an important role in plant development and could regulate gene expression at the transcriptional level such that the plants maintained normal physiological activity under stress [51]. The whole genes mainly clustered in ARR-B TFs, which played an important role in plant stress defense and development according to positive regulation in the phosphorelay-mediated cytokinin signal transduction [52]. AP2/EREBP (ethylene-responsive element-binding proteins), GRAS (gibberellin), and ARF (auxin) played an important role of plant hormones in abiotic stress responses and also gathered under different P treatments [53]. NAC, bHLH, WRKY, and bZIP, as the larger family of TFs, regulated plant stress, development, metabolism, and some other pathways, and also responded to P deficiency [54]. FAR1, MADS, and ABI3VP1 were more associated with plant growth and light signal transduction [31]. Some of these transcriptions also took part in other stresses [55]. The analysis of DEGs showed that 17 TFs were predicted and belonged to ARR-B, bHLH, GRAS, MADS, and NAC (Table S13). Zhao et al. studied the response of growth characteristics and endogenous hormones of *Sophora davidii* to low-P stress. Five phytohormones (abscisic acid, cytokinin, strigolactone, indole-3-acetic acid, and gibberellin) were regulated by P deficiency in the leaf samples [56]. Han et al. showed that the MADS TF gene (*TaMADS2-3D*) regulated phosphate starvation responses in plants [57]. NAC TFs also underwent intensive posttranslational regulation, including ubiquitinization, dimerization, phosphorylation, or proteolysis [58]. These TFs participated in regulating the P deficiency. Except for these TFs, the other six TFs were clustered in root DEGs (Table S11). Lei et al. also found that AP2-EREBP and bHLH TFs were among the most significantly differentially regulated genes identified under both Pi-sufficient and Pi-deficient conditions [59]. C2C2-CO-like and C2C2-Dof belonged to zinc finger protein, which could be involved in the geotropic growth of roots, and GRAS TFs also influenced the development in roots [60]. Further, P primarily acted on the roots. TIFY, WRKY, and HSP all reportedly regulated the plant growth under P deficiency. A large number of ARR-B TF-related genes were found in both leaves and roots. Therefore, the TF family was more closely related to P regulation. All of these gene clusters also revealed that a large number of TFs played an important role in improving the ability of crops to resist P starvation during growth and development.

To find out the main regulated genes under P deficiency in hulless barley, 19 genes were analyzed for their expressions by RT-PCR, which all had significant expression differences and good repeatability of each sample. Some of these DEGs participated in carotenoid biosynthesis (*HORVU0Hr1G017690*), arginine and proline metabolism (*HORVU7Hr1G090410*), and some abiotic stresses (*HORVU3Hr1G007500*, *HORVU3Hr1G086500*, *HORVU6Hr1G077710*, *HORVU6Hr1G082360*, *HORVU6Hr1G082360*, and *HORVU7Hr1G049370*). *HORVU7Hr1G089910* responded to phosphate starvation [61], and some other genes did not have predicted function annotation in the GO term and KEGG pathway. Thus, we selected two different low P concentrations compared with normal concentrations to identify the expression patterns of these genes. The expression patterns showed a few differences compared with those in the RNA-seq data, but a large number of genes exhibited a similar tendency. The results indicated that P deficiency could influence a lot of pathways, and the regulation of abiotic stress, heat stock, and phosphate starvation were normally influenced in these pathways. *MSTRG.19126* and *MSTRG.33383*, as new genes, were significantly upregulated under P deficiency in leaf samples, and low-P treatment also induced carotenoid biosynthesis, phosphate starvation, and arginine and proline metabolism in the roots. The expressions of *HORVU6Hr1G065240* and *HORVU1Hr1G000440*, which had no annotation information, also showed significant differences at different P concentrations. The functional and regulatory mechanisms require further experimental verification.

## 5. Conclusions

According to the RNA-seq results, we analyzed the relationship between the whole gene transcriptional processes and P deficiency response in hulless barley. The results primarily indicated that the regulatory genes participated in some pathways, including photosynthesis, amino acid biosynthesis, glycolysis, glycerolipid metabolism, carotenoid biosynthesis, and flavonoid biosynthesis. Oxidative phosphorylation and some TFs, which were related to phytohormones, could influence the transport and accumulation of P in the leaf and root samples under P deficiency. Some DEGs were enriched in phytohormone biosynthesis, photosynthesis, and some other transports, limiting the development under P deficiency. The present study enhanced the knowledge of the enrichment of gene networks and regulatory elements under P deficiency and provided a way for future research on P-use efficiency in hulless barley.

## Data Availability

No new data were created or analyzed in this study. Data sharing is not applicable to this article.

## Supplementary Materials

The following supporting information can be downloaded at https://www.mdpi.com/article/10.3390/life14070904/s1, File S1: Object IDs and corresponding URLs; Table S1: Difference in the phenotype, endogenous content, and enzymatic activity under P deficiency; Table S2: Statistics of RNA-seq data filtering and base information; Table S3: Reference gene coverage and gene alignment region; Table S4: Novel genes; Table S5: Gene and DEG expression in leaf and root samples; Table S6: Transcription factor information in whole genes and DEGs; Table S7: Level 2 GO terms in leaf and root samples; Table S8: Top 20 GO annotations in DEGs; Table S9: KEGG pathways in leaf and root samples; Table S10: Gene set enrichment analysis in leaves and roots; Table S11: DEG GO annotation under twofold difference expression; Table S12: DEG KEGG pathway under twofold difference expression; Table S13: Transcription factors under twofold difference expression; Table S14: Primer sequences using qRT-PCR; Table S15: The database of qRT-PCR results in 19 genes; Table S16: the expression pattern of all samples; Figure S1: Transcription factor family members in whole genes; Figure S2: GO annotation in DEGs in leaves and roots; Figure S3: KEGG pathway annotation in DEGs in leaves (A) and roots (B); Figure S4: the PCA of relationship between root treatment samples.
