# Supplementary material for: Comparative Analysis of Hulless Barley Transcriptomes to Regulatory Effects of Phosphorous Deficiency
Source: Life (Basel). 2024 Jul 19;14(7):904. doi: 10.3390/life14070904 (PMC11278117; doi:10.3390/life14070904)
Supplement: Supplementary file 1 [file life-14-00904-s001.zip › File S1.docx]

**Object IDs and corresponding URLs**

38060350 (WG1): https://www.ncbi.nlm.nih.gov/sra/38060350

38060351 (WG2): https://www.ncbi.nlm.nih.gov/sra/38060351

38060352 (WG3): https://www.ncbi.nlm.nih.gov/sra/38060352

38060353 (WL1): https://www.ncbi.nlm.nih.gov/sra/38060353

38060354 (WL2): https://www.ncbi.nlm.nih.gov/sra/38060354

38060355 (WL3): https://www.ncbi.nlm.nih.gov/sra/38060355

38060356 (ZG1): https://www.ncbi.nlm.nih.gov/sra/38060356

38060357 (ZG2): https://www.ncbi.nlm.nih.gov/sra/38060357

38060358 (ZG3): https://www.ncbi.nlm.nih.gov/sra/38060358

38060359 (ZL1): https://www.ncbi.nlm.nih.gov/sra/38060359

38060360 (ZL2): https://www.ncbi.nlm.nih.gov/sra/38060360

38060361 (ZL3): https://www.ncbi.nlm.nih.gov/sra/38060361
