# Supplementary figures and images for: Comparative Analysis of Hulless Barley Transcriptomes to Regulatory Effects of Phosphorous Deficiency
Source: Life (Basel). 2024 Jul 19;14(7):904. doi: 10.3390/life14070904 (PMC11278117; doi:10.3390/life14070904)

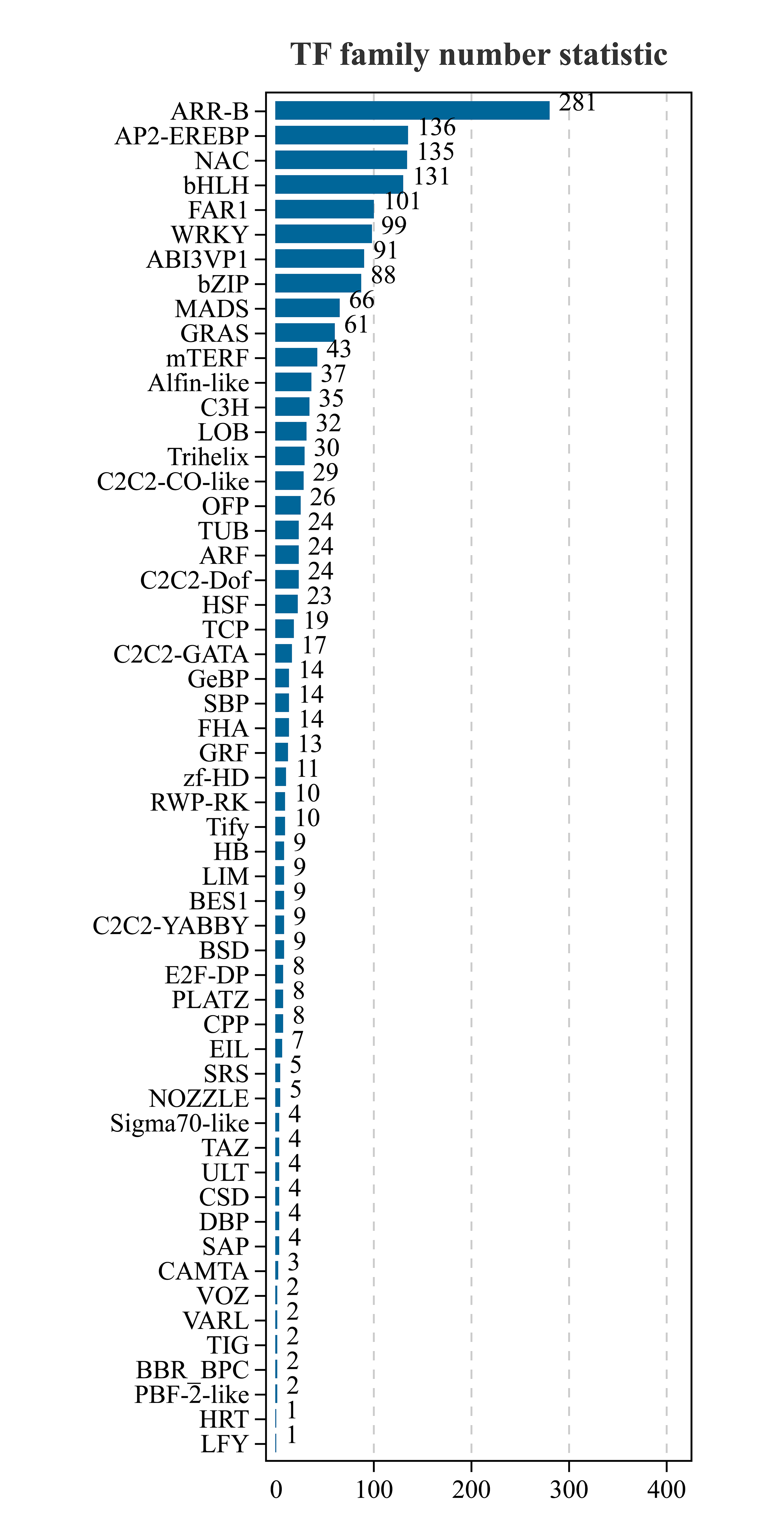

Supplement: Supplementary file 1 [file life-14-00904-s001.zip › Figure S1.png]

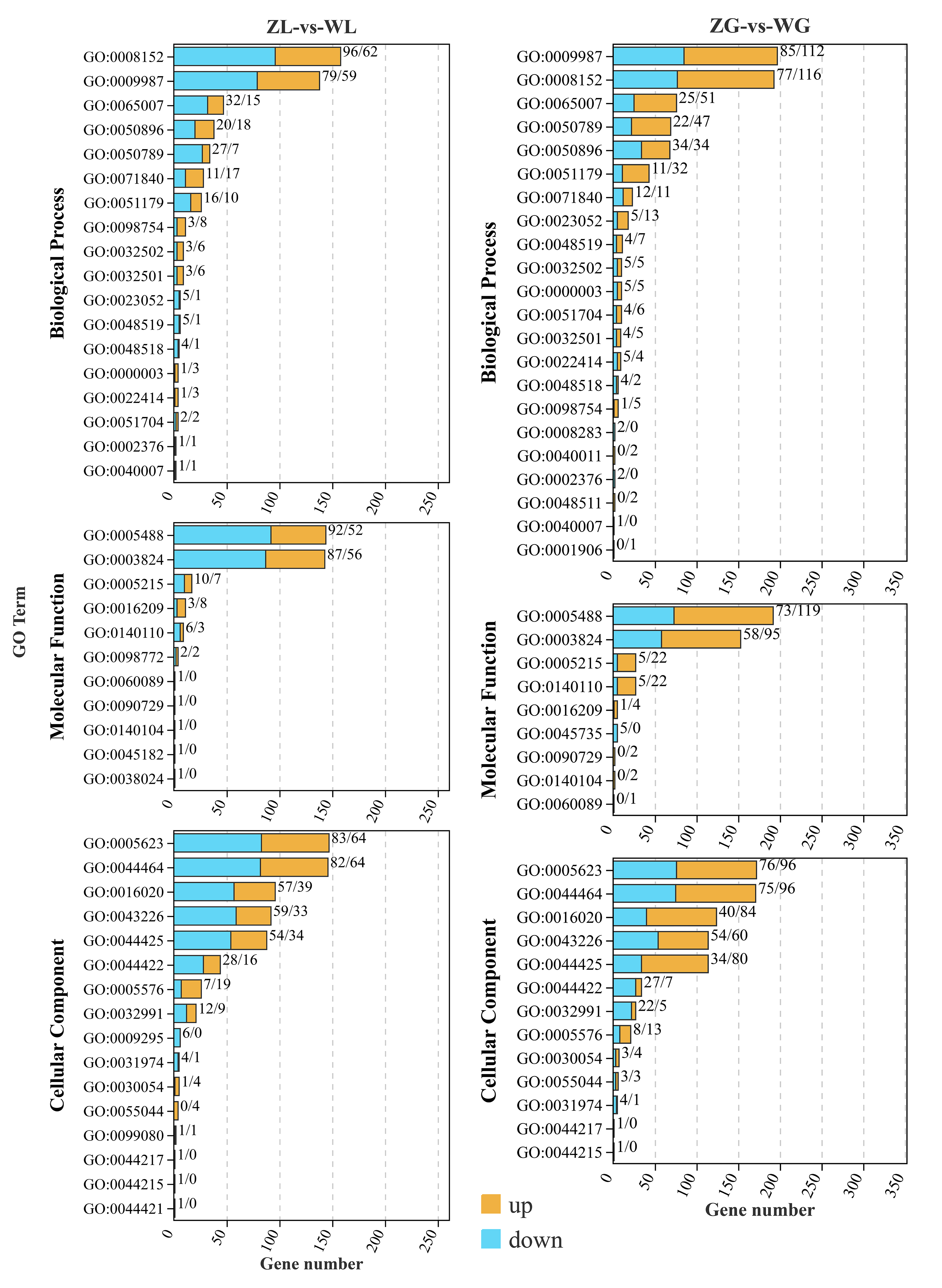

Supplement: Supplementary file 1 [file life-14-00904-s001.zip › Figure S2.png]

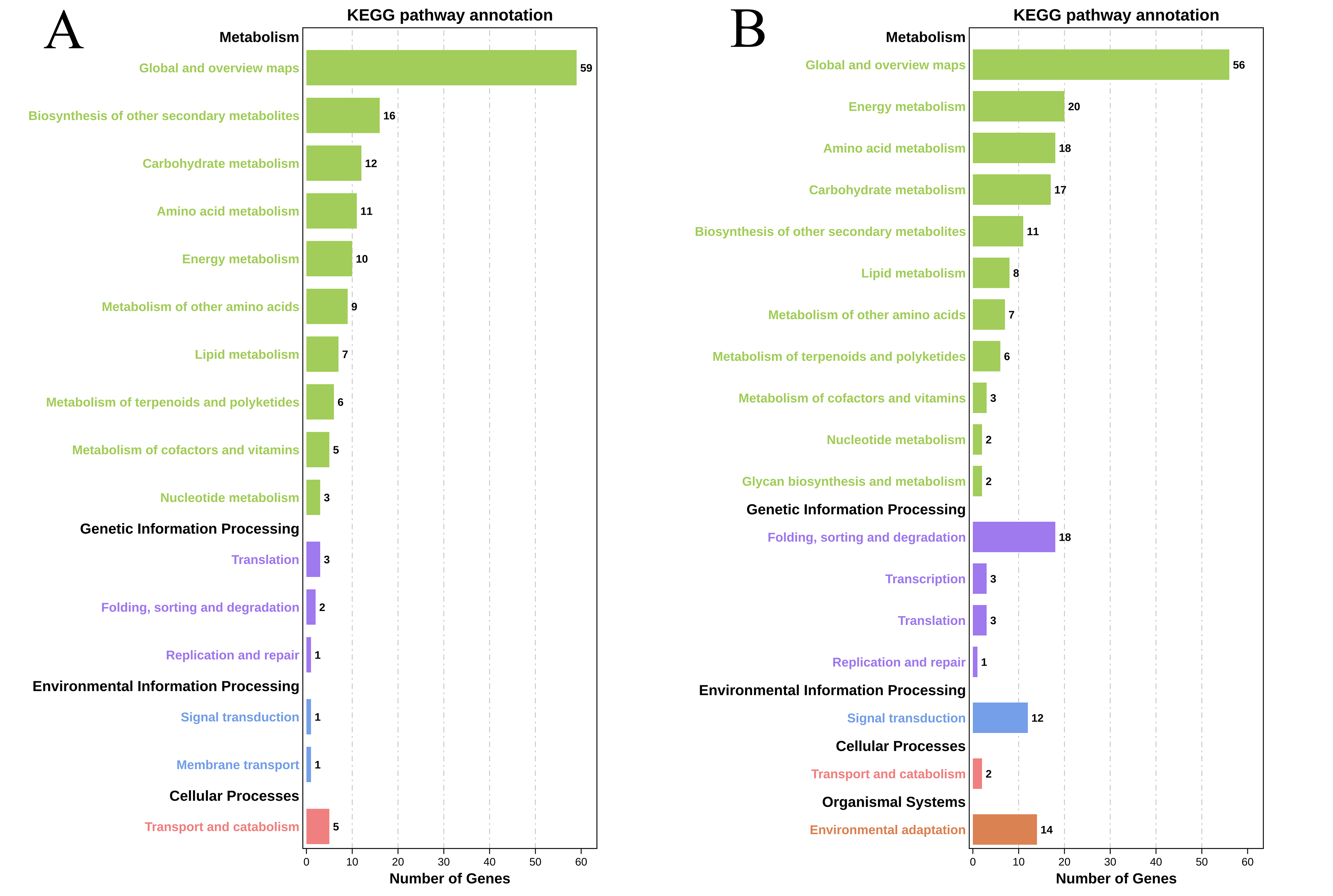

Supplement: Supplementary file 1 [file life-14-00904-s001.zip › Figure S3.png]

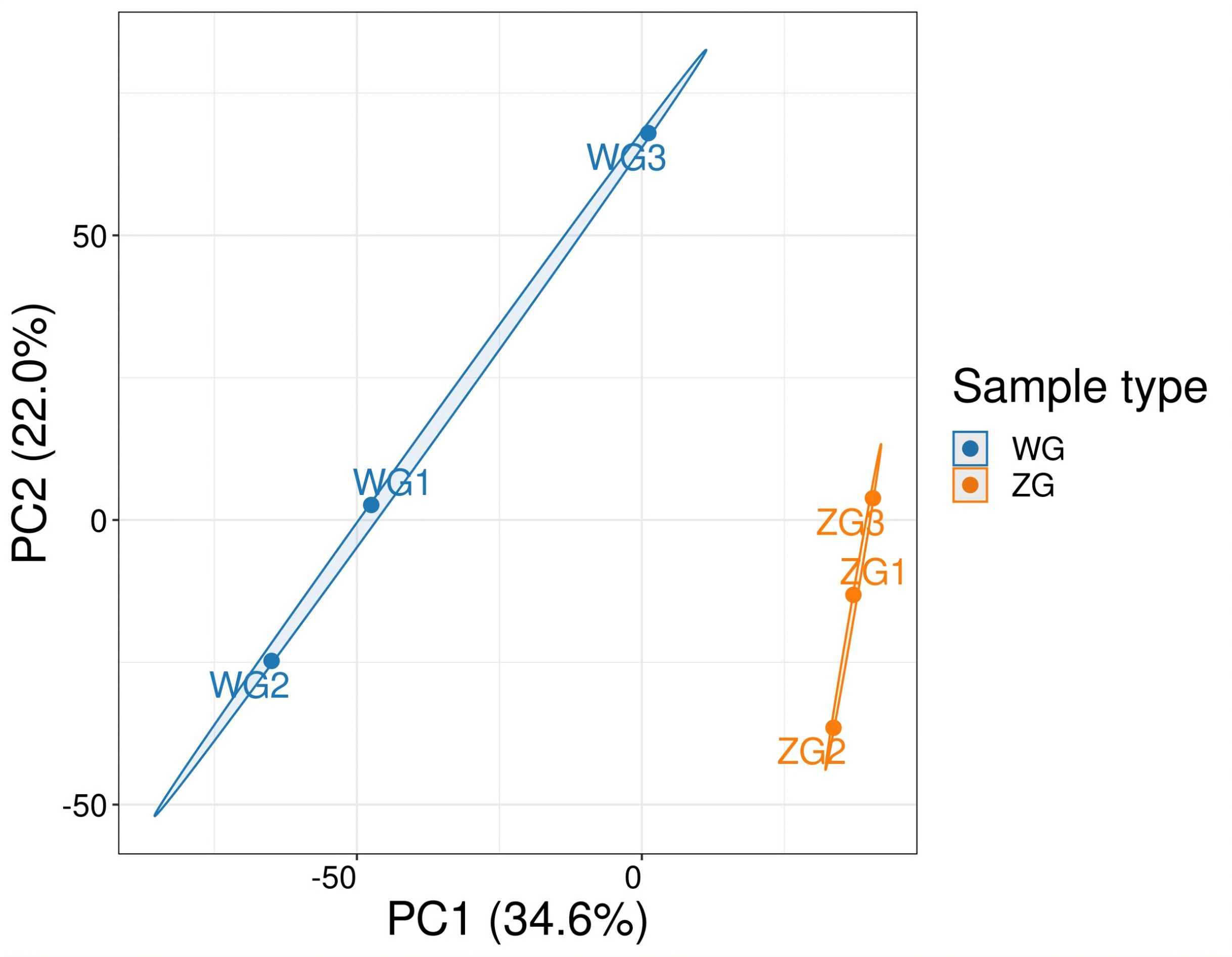

Supplement: Supplementary file 1 [file life-14-00904-s001.zip › Figure S4.png]
